# The aging clock: to ‘BMAL'icious toward learning and memory

**DOI:** 10.18632/aging.100144

**Published:** 2010-05-07

**Authors:** Jason R. Gerstner

**Affiliations:** Department of Genetics, University of Wisconsin-Madison, Madison, WI 53706, USA

**Keywords:** Kondratova et al. Circadian clock proteins control adaptation to novel environment and memory formation. Aging, 2010; this issue

Brain and Muscle Arnt-Like 1 (BMAL1), also known as MOP3 or ARNT3, is a basic helix-loop-helix (bHLH)-PAS domain-containing transcription factor that is necessary for the generation of circadian rhythms, and has been implicated in aging.  New work shows that BMAL1, or its binding-partner CLOCK, are needed for the generation of new memories.  These data suggest a novel molecular link between the processes of circadian rhythms, aging, and memory formation.

It is well known that the process of aging is associated with cognitive decline [1], and disruption in the ability to sleep [2]; recently these features of aging have been shown to be correlated [3].  However, common molecular players that are involved in mediating these processes have not been well characterized.  Recent work has begun to establish common molecular mechanisms in the processes of circadian rhythms and memory formation [4], but their relationship to aging so far has not been determined.  Now a study by Kondratova et al. [5] in this issue of *Aging* provides evidence for a core component of the circadian clock, BMAL1, which has previously been shown to significantly influence lifespan [6], in the regulation of learning and memory behavior.

**Figure 1.**
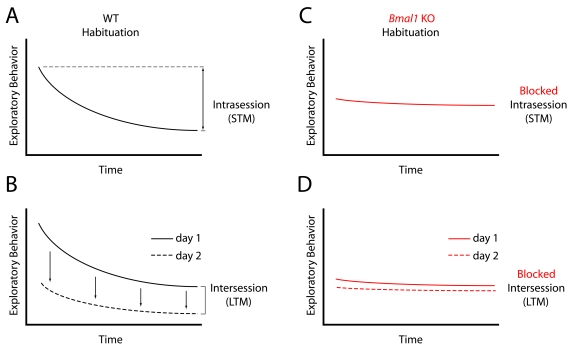
(**A**) Wild-type (WT) mice exhibit a decrease in exploratory behavior in a novel environment over time, called "Intrasession" habituation, a form of short-term memory (STM). (**B**) Upon reintroduction to the same environment 24 hours later, WT mice have a further decrease in exploratory behavior, attributable to remembering the previous experience.  This is called "Intersession" habituation, and is considered a form of long-term memory (LTM).  (**C**) *Bmal1* KO mice fail to display normal Intrasession, and  (**D**) Intersession habituation, suggesting deficits in both STM and LTM.

Kondratova et al. [5] examined the role of circadian genes in adaptation, by studying the exploratory behavior and habituation to novelty in various circadian mutant mice, using the open field paradigm.  When wild-type (WT) mice are placed in an open field, their novel experience generates an increase in exploratory behavior that begins to decline over time, as the animal remembers the environment (*intra*session habituation), and is considered a form of short-term memory (Figure 1A).  When WT mice are then placed in this same environment on subsequent days, they exhibit further reductions in exploratory behavior (*inter*session habituation), which is associated with the formation of long-term memory (Figure 1B).  Surprisingly, *Bmal1* knock-out (KO) mice display an inability in both intra- and intersession habituation (Figure 1C, D), suggesting that *Bmal1* gene expression is necessary for both short- and long-term memory.

**Figure 2.**
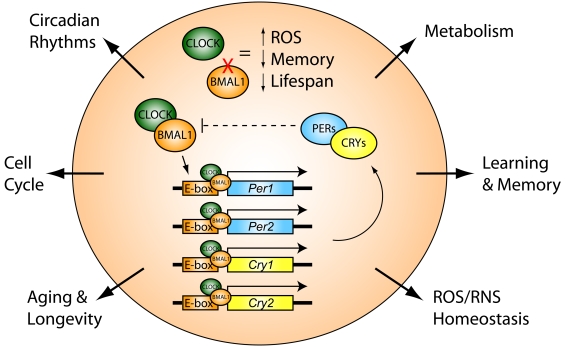
Core components of the circadian transcriptional clock. Brain- and Muscle ARNT-like protein (BMAL1) heterodimerizes with CLOCK protein to bind E-box motifs in the promoter regions of downstream target genes, such as *Period* (*Per 1,2*) and *Cryptochrome* (*Cry1,2*) genes.  PERIOD proteins (PERs) heterodimerize with CRYPTOCHROME proteins (CRYs) in order to inhibit CLOCK:BMAL1, thus closing an autoregulatory negative-feedback loop.  Blocking activity of CLOCK:BMAL1 in *Bmal1* knock-out mice disrupts normal circadian rhythms, and increases reactive-oxygen species (ROS), while concomitantly decreasing memory and lifespan.  Circadian clock output regulates a variety of biological and physiological processes, including circadian rhythms, metabolism, learning and memory, ROS/reactive nitrogen species (RNS) homeostasis, aging and longevity, and the cell cycle.

Next, the authors examined the effects on habituation in mice carrying a mutation for the BMAL1 binding partner CLOCK (*Clock/Clock*).  CLOCK protein forms a heterodimer with BMAL1, and binds to E-box regulatory sequences, in order to drive transcription in the promoter region of downstream target genes, such as *Period* (*Per1,2*) and *Cryptochrome* (*Cry1,2*), which are also intimately involved in the regulation of circadian rhythms [7-9] (Figure 2). Interestingly, *Clock/Clock* mice display normal intrasession habituation, but have significantly reduced intersession habituation, suggesting that CLOCK, like BMAL1, is necessary for long-term memory formation.  While *Clock/Clock* mice retain normal short-term memory, the fact that these mice are also deficient in long-term memory formation provides further evidence supporting a role for circadian machinery in the regulation of long-term memory.

Since both *Bmal1* KO and *Clock/Clock* mice display a reduction in intersession habituation, Kondratova et al. [5] also examined mice deficient in *Cry1,2*  (*Cry1,2* KO) in the open field paradigm.  CRY proteins (CRYs) heterodimerize with PER proteins (PERs) to inhibit CLOCK:BMAL1 -mediated transcription, thus generating an autoregulatory negative-feedback loop in the circadian clock (Figure 2).    Amazingly, *Cry1,2* KO mice not only exhibit both intra- and intersession habituation, but seem to have accelerated habituation; this suggests that *Cry1,2* KO mice may have improved short- and/or long-term memory.  This interpretation would make sense, given that the *Cry1,2* KO mice likely *dis*-inhibit CLOCK:BMAL1 transcriptional activity, thereby providing a net increase in downstream target gene expression, such as *Per1,2*.  Overexpression of *Per* in the fruit fly *Drosophila melanogaster* has already been shown to enhance long-term memory in courtship conditioning, while *Per* null flies have significantly impaired memory [10].  Together, these data support a mechanistic relationship for circadian genes in the processes of learning and memory.

Imbalance of reactive oxygen species/nitrogen species (ROS/RNS) homeostasis is associated with aging and cognitive decline [1].  In the current study, Kondratova et al. [5] show that ROS homeostasis is also altered in the brains of *Bmal1* KO mice.  While levels of ROS do not seem to vary with time-of-day, on average, *Bmal1* KO mice have a significant increase in brain ROS as compared to WT, corroborating previous results that have shown increased ROS levels in *Bmal1* KO mice with accelerated aging [6]. Interestingly, a recent report found an accumulation of oxidative damage with a significant reduction in lifespan following oxidative stress in *Per* null flies, as compared to controls [11].  These data support a central role for clock genes in regulating ROS homeostasis, and suggest disruption of circadian pathways results in excessive production of ROS and chronic oxidative stress in the brain.

The circadian clock has been shown to regulate a variety of biological and physiological processes, including sleep/wake rhythms, the cell cycle, metabolism, and aging; and its dysfunction has broad implications in human health [9, 12, 13]. The current study offers further support for the critical involvement of circadian molecules in the regulation of cognitive processing and ROS/RNS homeostasis (Figure 2), providing a molecular link between sleep/wake rhythms, aging, and memory.  Loss of the normal sleep/wake cycle is a primary cause of institutionalization for dementia, and is even thought to be a contributing factor, and/or preclinical sign of neurodegenerative disease, such as Alzheimer's and Huntington's disease [14-16]. In a mouse model of Huntington's disease, which has progressive disruption of sleep/wake rhythms with age [17], reversal of the cognitive decline and survival rate can be achieved by a timed delivery of the benzodiazepine alprazolam, which reinstitutes a daily cycle of sleep [18, 19]. Observations such as these warrant further study into the functional relationships of other molecular players involved in the circadian clock, especially those that have a role in metabolism, such as PPARs, Rev-erbα,  and  Sirtuins [13, 20, 21].  Discovering ways that the clock machinery regulates aging, metabolism, the cell cycle, and memory formation (Figure 2) will undoubtedly offer novel therapeutic strategies for the treatment of age-related diseases, such as neurodegeneration, cognitive- and sleep-related disorders, and cancer.
